# Increased CX3CL1/CX3CR1 Axis Is Related to Atherosclerosis in Subjects with Familial Combined Hyperlipidaemia, Which Is Modulated by Insulin Resistance but Not by Sex

**DOI:** 10.3390/biomedicines13102378

**Published:** 2025-09-28

**Authors:** Elena Jiménez-Martí, Clara Espinosa-Bellido, Blanca Alabadi, Gema Hurtado-Genovés, Antonio Enrique-Medina, Susana Martín-Vañó, Víctor Casas, Eduardo A. Cortés Nadal, José T. Real, Herminia González-Navarro, Sergio Martínez-Hervás

**Affiliations:** 1 Department of Biochemistry and Molecular Biology, University of Valencia, 46010 Valencia, Spain; elena.jimenez@uv.es; 2 INCLIVA Biomedical Research Institute, 46010 Valencia, Spain; balabadi@incliva.es (B.A.); gemahurge@gmail.com (G.H.-G.); susana.martin@uv.es (S.M.-V.); jtreal@uv.es (J.T.R.); 3 Service of Endocrinology and Nutrition, Hospital Clínico Universitario of Valencia, 46010 Valencia, Spain; claraespibellido@gmail.com (C.E.-B.); antonioenriquemedina@gmail.com (A.E.-M.); 4 Faculty of Medicine, University of Valencia, 46010 Valencia, Spain; vcasasbenavent@gmail.com (V.C.); eduardo.nadal@gmail.com (E.A.C.N.); 5 Department of Medicine, University of Valencia, 46010 Valencia, Spain; 6 CIBER de Diabetes y Enfermedades Metabólicas Asociadas (CIBERDEM), Institute of Health Carlos III, Minister of Science, Innovation and Universities, 28029 Madrid, Spain

**Keywords:** atherosclerosis, familial combined hyperlipidaemia, insulin resistance, CX_3_CL1/CX_3_CR1 axis, inflammation

## Abstract

**Background**: A major factor in the development of atherosclerosis is the presence of a chronic inflammatory state. The CX_3_CL1/CX_3_CR1 axis has been implicated in the development of atherosclerosis and cardiovascular disease, but until now, scarce data are available regarding the influence of the CX_3_CL1/CX_3_CR1 axis in familial combined hyperlipidaemia (FCH). Since FCH is associated with a high risk of cardiovascular disease, the objective of the present study was to assess the presence of alterations in the CX_3_CL1/CX_3_CR1 axis in patients with FCH and to evaluate the influence of insulin resistance (IR) and sex. **Methods**: A cohort of 47 subjects with FCH and 38 control subjects was included. We measured the lipid profile, glucose, and insulin levels in plasma, circulating blood CX_3_CL1 levels, and CX_3_CR1 mRNA expression. Carotid IMT and the presence of atheroma plaques were also evaluated. **Results**: FCH subjects showed significantly higher activation of the CX_3_CL1/CX_3_CR1 axis than controls. In addition, FCH individuals with IR showed the worst profile of inflammation status, higher carotid IMT, and a higher prevalence of atherosclerotic plaque compared to controls and FCH patients without IR. However, sex did not influence the CX3CL1/CX3CR1 axis. **Conclusions**: FCH patients showed an increased CX_3_CL1/CX_3_CR1 axis, which was positively correlated with IR, but not with sex. These data could partially explain the increased risk of cardiovascular events in primary dyslipidemic patients.

## 1. Introduction

Familial combined hyperlipidemia (FCH) is a highly prevalent primary dyslipidemia (1–3% in the general population), frequently associated with insulin resistance (IR), characterized by the presence of atherosclerosis and premature cardiovascular disease [1].

The risk of developing atherosclerosis is not uniform. Previous studies have shown differences between women and men in cardiovascular risk factors and cardiovascular disease incidence [2]. However, traditional risk factors do not fully explain that risk. A major factor in the development of atherosclerosis is the presence of a chronic inflammatory state. Different factors, including sex, influence the inflammatory state that promotes the formation and progression of atherosclerotic plaques [3]. Various studies suggest that women could have higher levels of inflammatory markers than men and a lower anti-inflammatory response [4].

Multiple molecular and cellular mechanisms of inflammation have been involved in atherosclerosis. One of the axes implicated in the development of atherosclerosis and cardiovascular disease is the CX_3_CL1/CX_3_CR1 axis [5,6]. Fractalkine/CX_3_CL1 is a transmembrane protein widely expressed in human cells, including immune cells. CX_3_CL1 expression is markedly upregulated in human endothelial cells during the inflammatory process. Furthermore, the CX_3_CL1/CX_3_CR1 axis has been involved in crucial steps of the atherogenic process [7,8].

Previous studies have shown that FCH is associated with an enhanced inflammatory status, especially in the presence of IR [9,10,11]. IR has been shown to increase plaque vulnerability by overexpressing the CX_3_CL1/CX_3_CR1 axis [12], which is one of the possible factors linking diabetes or metabolic syndrome with cardiovascular disease. However, there are no data available on this axis in patients with FCH. In this sense, we hypothesized that, in FCH, a genetic model of dyslipidemia with a high risk of developing cardiovascular disease, there could be alterations in the CX_3_CL1/CX_3_CR1 axis modulated by the presence of insulin resistance and sex, which could explain why some are more prone to developing cardiovascular disease.

## 2. Materials and Methods

### 2.1. Study Design and Population

Subjects were recruited over a period of 1 year at the Lipid Unit of the Hospital Clinico Universitario of Valencia using opportunistic method. We included 47 subjects with FCH and 38 control subjects who underwent an annual regular physical check-up.

The diagnosis of FCH was based on the presence of dyslipidaemia (LDL cholesterol and/or triglycerides (TG) levels exceeding the 90th percentile of our population by age and sex), elevated plasma levels of apolipoprotein B (apoB) > 120 mg/dL, and first-degree relatives with variable lipid phenotypes (IIa, IIb, or IV) or a family history of premature atherosclerosis. Furthermore, one of the following additional features must be present: TC > 240 mg/dL, LDL-C > 160 mg/dL, TG > 150 mg/dL, or HDL-C < 50 mg/dL or <40 mg/dL for females and males, respectively. The absence of xanthomas was also considered [13].

The subjects included in the control group were nondiabetic and normolipidemic (TC concentration < 200 mg/dL, TG < 150 mg/dL, and apo B < 100 mg/dL), with no family history of dyslipidaemia, cardiovascular disease, or diabetes.

Exclusion criteria were patients with chronic inflammatory diseases (both digestive and rheumatic), plasma levels of high-sensitivity C-reactive protein (hsCRP) > 10 mg/L, and any infectious or inflammatory episode within the previous 2 weeks.

The project was approved by the Ethical Committee of the Hospital Clinico Universitario of Valencia (Ref 2019/059) on 12 September 2019. All participants gave written informed consent before their inclusion in this study.

### 2.2. Anthropometric Parameters

The parameters assessed were weight in kilograms (kg), height in metres (m), body mass index (BMI) in kg/m^2^, and waist circumference in centimetres (cm), measured at the midpoint between the anterior superior iliac spine and the lower costal margin, with the subject standing with arms in the anatomical position. Measurements were obtained using a tape measure graduated in cm.

Blood pressure was measured in the sitting position after a 10-minute resting period, with two separate measurements taken.

Abdominal circumference was measured in cm (at the point between the lower costal rim and the iliac crest) by the same investigator.

### 2.3. Biochemical Parameters

Venous samples were obtained after a 12-h overnight fast. Serum concentrations of TC, TG, HDL-C, apo B, glucose, and insulin were measured using standard methods, as previously described [14]. The LDL-C concentration was calculated according to the Friedwald equation. IR was calculated using HOMA, considering IR if HOMA ≥ 3.2, based on previous studies in the Spanish population [15].

### 2.4. CX_3_CL1 and CX_3_CR1 Determination

#### 2.4.1. Enzyme-Linked ImmunoSorbent Assay (ELISA) for CX_3_CL1 Detection

For the determination of circulating CX_3_CL1 blood levels, ELISA analysis was performed on plasma from heparinized (10 U heparin/mL) human whole blood samples obtained from patient groups using the Human CX3CL1/Fractalkine DuoSet ELISA (R&D Systems, Minneapolis, MN, USA). The procedure followed the commercial recommendations, and results were expressed in pMols/L, as previously described [12].

#### 2.4.2. Gene Expression Analysis via Quantitative Real-Time PCR (qPCR) for CX_3_CR1

Gene expression analysis was performed on RNA from peripheral blood mononuclear cells (PBMC) of patients, which were isolated from blood employing Ficoll-Hypaque (GE HealthCare, Thermo Fisher, Barcelona, Spain) density gradient centrifugation. The obtained PBMC were used to obtain RNA with TRIzol Reagent (Invitrogen, Carlsbad, CA, USA) following the indicated procedure. RNA purity and concentration were determined by the A260/A230 ratio and A260/280 ratio using a NanoDrop spectrophotometer (NanoDrop 2000, Thermo Scientific). RNA purity and quality were determined by the A260/A230 ratio and A260/A280 ratio using a NanoDrop spectrophotometer (Thermo Scientific Nano Drop 2000). The A260/A230 and A260/A280 ratios ranged from 1.8 to 2.0, and RNA concentrations were 300–700 ng/µL per sample. RNA (0.5–1 μg) was retrotranscribed using the Maxima First Strand cDNA Synthesis kit (Fermentas, Thermo Fisher Scientific, Madrid, Spain) in a mix consisting of 500 ng of RNA in a volume of 20 µL. For amplification of the *CX3CR1 gene* and the endogenous *GAPDH gene*, 25 ng of retrotranscribed cDNA was employed in a 20 µL mix with 10 µL of HiGreen with Luminars Color HiGreen High ROX qPCR Master MIX K0362 (Fermentas, Thermo Fisher Scientific, Madrid, Spain) and a final concentration of 30 µM of each forward and reverse primer. Reactions were run on a thermal Cycler 7900 Fast System, and results were analyzed with the software provided by the manufacturer (Applied Biosystems, Life technologies, Madrid, Spain). The amplification of the genes of interest was monitored in real time through the detection of the fluorescence of a green fluorescent probe intercalated into the amplified DNA fragments during each cycle. The PCR protocol used was as follows: 40 cycles were run, followed by a cycle of melt curve stage, consisting of hold stage (1× incubation for 2 min at 50 °C, 1× incubation for 10 min at 95 °C), PCR stage (40× incubation for 15 s at 95 °C and 1 min at 60 °C), and melt stage (1× incubation for 10 min at 60 °C, followed by 15 s at 95 °C).

To quantify gene expression, the relative quantification method was used, which requires standards and allows determining the magnitude of physiological changes in the expression levels of a gene compared to a reference gene. Quantification was carried out using the values of the threshold cycle (Ct), applying the 2^−ΔΔCt^ method [16], which establishes the following formula to calculate relative expression:

Relative quantification = 2^−(ΔCt sample − ΔCt reference)^ → RQ = 2^−ΔΔCt^.

This approach allows correcting possible technical variations (such as partial RNA degradation, variable efficiency in reverse transcription, or pipetting errors) using an internal control (the endogenous gene), thus providing a value proportional to the total amount of mRNA of each gene. This allows comparison of expression levels between different genes or between different experimental groups. According to this procedure, the mRNA levels of each gene were normalized against the mRNA levels of the reference gene GAPDH. Gene expression was expressed relativized to control samples from control subjects (assigning RQ = 1 to control samples in each experiment, according to the formula indicated above).

To detect the gene of interest, *CX3CR1*, and the endogenous control *GAPDH*, the corresponding primers were designed using the primer express program and were as follows: human *GAPDH* Fw 5′-ACCACAGTCCATGCCATCAC-3′ and Rv 5′-TCCACCACCCTGTTGCTGTA-3′; human *CX3CR1*: FW 5′-CGTCATCAGCATTGATAGGTACCT-3′ and Rv 5′-CTGCACGGTCCGGTTGTT-3′. Expression was displayed normalized to endogenous mRNA levels and relative to a larger control group employed for a larger clinical investigation of the Clinical Service.

### 2.5. Carotid Ultrasound

High-resolution carotid and femoral ultrasonography was performed using the 8 to 12 MHz transducer of a GE Logiq F6 ultrasound scanner (GE Healthcare, Chicago, IL, USA). Carotid examination was performed with the subjects in the supine position, with the head turned 45° away from the side being explored. Four predetermined segments were evaluated on both sides: common carotid (1 cm proximal to the carotid bulb), carotid bulb (1–2 cm), and internal and external carotid (1 cm distal to the bifurcation). Evaluation was performed bilaterally in 3 different projections (right side: 90, 120, and 150°; left side: 210, 240, and 270°). Intima–media thickness (IMT), defined as the distance between the carotid lumen–intima interface and the media–adventitia interface of the distal wall, was determined in longitudinal sections of the region anterior to the bifurcation of the common carotid artery (1 cm). Atheroma plaque was considered in the presence of a focal thickening of more than 50% of the surrounding vessel wall or an IMT (intima–media thickness) greater than 1.5 mm that protruded into the adjacent lumen [17]. All examinations were performed by the same investigator (SM-H), trained in performing vascular ultrasounds and always following the identical protocol. The coefficient of variability was previously studied in 20 subjects and was 5.2% for the mean IMT of both common carotids.

### 2.6. Statistical Methods

Data were analyzed using the Statistical Package for the Social Sciences (SPSS 28 for Windows; SPSS, Chicago, IL, USA). Results are expressed as mean ± standard deviation for quantitative variables and as percentages and/or total number for qualitative variables.

Normal distribution was evaluated for each variable. To compare normally distributed quantitative variables between groups, Student’s *t*-test and ANOVA test were used (2 or more variables, respectively). For non-normally distributed variables, the Mann–Whitney test and the Kruskal–Wallis test were used (2 or more variables, respectively). To correct confounding factors in some comparison studies, ANCOVA analysis was used. To compare the qualitative variables between groups, the Chi-square test was used, or the Fisher test when the number was less than 5. Bivariate correlations between variables were studied with the Pearson test for variables with a normal distribution and the Spearman test for variables without a normal distribution. *p*-values were two-tailed. Differences were considered statistically significant if *p*-value was less than 0.05.

## 3. Results

The characteristics of the subjects included in this study are shown in Table 1. There were no significant differences in age. However, the rest of the parameters, related to the presence of primary dyslipidaemia, showed significant differences because of the inclusion criteria of both groups. Those differences remained significant between controls and subjects with dyslipidaemia when the population was divided according to sex. However, we did not find differences between sexes when we analyzed each of the groups separately.

When we divided the subjects with FCH according to the presence of IR (Table 2), we found that FCH subjects with IR showed the worst metabolic profile compared to controls and non-IR FCH. Moreover, FCH with IR showed significantly increased circulating CX_3_CL1 protein and mRNA levels of CX_3_CR1 in PBMC, consistent with increased activation of the CX_3_CL1/CX_3_CR1 axis. FCH patients with IR also showed higher carotid IMT and a higher prevalence of atherosclerotic plaque. However, the CX_3_CL1/CX_3_CR1 axis did not show significant differences between non-IR FCH and controls. Furthermore, carotid IMT and the prevalence of atheroma plaque were similar in both groups, non-IR FCH and controls.

We also analyzed the influence of sex according to the presence of IR in each group, controls, and FCH (Table 3). Women and men with FCH and IR showed significant differences in all the clinical and biochemical parameters compared to women and men in the control group. Both sexes of subjects with FCH and IR also had significantly higher carotid IMT and a higher prevalence of atheroma plaque compared to women and men in the control group. However, the CX_3_CL1/CX_3_CR1 axis did not show significant differences between women and men in any of the three groups.

Bivariate correlations between the CX_3_CL1/CX_3_CR1 axis and the rest of the variables are shown in Table 4. Significant correlations were observed with metabolic disturbances associated with an insulin-resistance state. CX_3_CL1 levels were associated with carotid plaque, and CX_3_CR1 expression was associated with carotid IMT.

## 4. Discussion

Our results show that patients with FCH have increased expression of CX_3_CL1 and its receptor, CX_3_CR1, compared with healthy controls. Furthermore, the CX_3_CL1/CX_3_CR1 axis was significantly associated with higher carotid IMT and the presence of atheroma plaque. This association seems to be influenced by the presence of IR, which induces a worse metabolic profile, aggravates atherosclerosis, and upregulates the CX_3_CL1/CX_3_CR1 axis.

Inflammation is a critical pathway in the pathogenesis of atherosclerosis [18]. Many inflammatory axes have been implicated, with the CX_3_CL1/CX_3_CR1 axis being one of them [19]. Fractalkine is a unique chemokine that presents as a transmembrane protein in the endothelium or, after cleavage, as a soluble ligand that attracts leukocyte subsets expressing the corresponding receptor CX_3_CR1 [20]. This axis is involved in a critical step of the atherogenic process since it participates in the mononuclear cell attachment and the endothelial transmigration of monocytes and lymphocytes [21].

The role of the CX_3_CL1/CX_3_CR1 axis in atherosclerosis and cardiovascular disease has been demonstrated in different studies. This axis has been linked to an increase in vulnerable atherosclerotic plaques in mice and to enhanced coronary artery disease in humans. Inhibition of CX_3_CR1 in animal models has been shown to lead to the prevention of atherosclerosis, showing a dramatic reduction in plaque size and composition, consistent with more stable features [21,22,23]. Moreover, patients with unstable angina and unstable plaque have shown higher CX_3_CL1 levels and increased expression of CX_3_CR1 [24,25]. Our results are in accordance with previous studies. We found that subjects with FCH, a genetic model of high cardiovascular risk, showed increased hsCRP levels and an increased CX_3_CL1/CX_3_CR1 axis. Furthermore, these subjects showed significantly higher IMT and a higher percentage of atheromatous plaque, both significantly correlated with the CX_3_CL1/CX_3_CR1 axis.

FCH is characterized by the presence of IR [26]. IR is an independent cardiovascular risk factor that has been associated with atherosclerosis and cardiovascular disease. The relationship between these two conditions is complex and bidirectional, with IR exacerbating atherosclerosis and vice versa. Moreover, IR is closely interconnected with inflammation, often creating a vicious cycle that exacerbates metabolic dysfunction and increases the risk of type 2 diabetes and cardiovascular disease [27]. In our study, although patients with FCH showed worse metabolic profiles, a higher prevalence of carotid abnormalities, and an increased CX_3_CL1/CX_3_CR1 axis, patients with FCH and IR were those who showed the worst association with cardiovascular risk factors, the highest carotid IMT, a higher prevalence of atheromatous plaque, and the highest expression of the CX_3_CL1/CX_3_CR1 axis. We have previously demonstrated that IR stimulates the CX_3_CL1/CX_3_CR1 axis, inducing vascular smooth muscle cells apoptosis, which is reflected in vivo by the development of unstable plaque features. Therefore, IR and the CX_3_CL1/CX_3_CR1 axis could be two important mechanisms driving accelerated atherosclerosis in these subjects.

Sex-related differences in morbidity and mortality rates from cardiovascular disease have been observed [28]. Previous studies suggest that women have a differential inflammatory response to various disease states, which increases their risk for cardiovascular disease [4]. CRP levels have been shown to be elevated in women compared to men [29,30]. Furthermore, autoimmune disorders are more prevalent in women [31]. In addition, women with an autoimmune disease are at increased risk of developing cardiovascular disease at younger ages [32]. However, there are relatively limited data that rigorously address the role of sex as a biological variable in the inflammatory processes associated with the development of atherosclerosis. In fact, in our study, which included 52.1% of women, we found no significant differences in the CX_3_CL1/CX_3_CR1 axis. However, the sample size included was very small; thus, the possible influence of sex cannot be ruled out.

Therefore, based on previous data and on our results, understanding the role of the CX_3_CL1/CX_3_CR1 axis, as well as the possible mechanisms involved in modulating the atherosclerotic process, is of great interest, especially since it could be a potential way for reducing the risk of developing atherosclerosis and cardiovascular disease. Ongoing clinical trials, such as FRACTAL, will clarify the therapeutic potential role of fractalkine inhibition [16].

However, the present study also has important limitations. First, we included only a small sample, and the proportion of both sexes was not uniform across groups. Additionally, as this was a cross-sectional study, the relationships found can only be used as hypothesis-generating and cannot be interpreted as causal.

## 5. Conclusions

In conclusion, we evaluated the activation of the CX_3_CL1/CX_3_CR1 axis in subjects with FCH, a human genetic model of mixed dyslipidaemia and IR. The finding of increased levels of CX_3_CL1 and increased expression of CX_3_CR1 in asymptomatic FCH subjects, associated with the presence of atherosclerosis, especially in the presence of IR, suggests that this activation could be an important factor in the development of atherosclerosis and cardiovascular disease in patients with primary dyslipidaemia. Thus, CX_3_CL1/CX_3_CR1 axis activity could have prognostic value in addition to that of traditional risk factors. However, further prospective and interventional studies are needed to evaluate the impact of the CX_3_CL1/CX_3_CR1 axis and the impact of sex on the pathogenesis of cardiovascular diseases in affected populations.

## Figures and Tables

**Table 1:** Characteristics of the population according to diagnosis and sex.

	FCH Group			Control Group		
	Men (*n* = 21)	Women (*n* = 26)	All (*n* = 47)	Men (*n* = 18)	Women (*n* = 20)	All (*n* = 38)
Age (years old)	56.2 ± 7.2	58.4 ± 9.1	57.4 ± 8.3	53.1 ± 7.6	57.2 ± 9.2	55.3 ± 8.6
BMI (kg/m^2^)	29.8 ± 3.4 *	32.7 ± 6.6 *	31.4 ± 5.6 *	24.8 ± 3.2	25.7 ± 3.4	25.3 ± 3.3
Waist circumference (cm)	103.3 ± 9.8 *	100.3 ± 16.3 *	100.3 ± 16.3 *	88.9 ± 10.0	85.3 ± 9.5	86.9 ± 9.8
SBP (mmHg)	135.9 ± 9.6 *	138.9 ± 15.1 *	137.6 ± 12.9 *	120.5 ± 15.4	123.7 ± 18.3	122.2 ± 16.9
DBP (mmHg)	84.3 ± 7.2 *	88.0 ± 9.9 *	86.4 ± 8.9 *	73.6 ± 9.1	75.0 ± 10.5	74.4 ± 9.8
Glucose (mg/dL)	126.2 ± 36.8 *	111.0 ± 27.8 *	117.8 ± 32.7 *	89.3 ± 8.8	88.2 ± 7.7	88.7 ± 8.1
Insulin (μU/mL)	15.7 ± 6.8 *	15.2 ± 9.1 *	15.4 ± 8.1 *	6.2 ± 2.3	6.0 ± 2.6	6.1 ± 2.4
HOMA index	4.9 ± 2.6 *	4.3 ± 2.9 *	4.6 ± 2.8 *	1.4 ± 4.9	1.3 ± 0.6	1.3 ± 0.5
Total cholesterol (mg/dL)	226.9 ± 43.1 *	240.1 ± 47.2 *	234.2 ± 45.4 *	203.7 ± 16.7	212.0 ± 28.2	208.1 ± 23.6
HDL-C (mg/dL)	45.8 ± 10.1 *	58.1 ± 10.7	52.6 ± 12.1 *	55.6 ± 13.4	62.7 ± 9.6	59.3 ± 11.9
LDL-C (mg/dL)	141.9 ± 24.9	150.7 ± 39.5	146.8 ± 33.8 *	134.2 ± 16.4	135.2 ± 24.9	134.8 ± 21.1
Triglycerides (mg/dL)	213.7 ± 106.9 *	170.6 ± 88.8 *	189.9 ± 98.6 *	80.6 ± 26.2	84.5 ± 23.2	82.6 ± 24.4
Apo B (mg/dL)	114.6 ± 20.4 *	111.5 ± 24.3 *	112.8 ± 22.4 *	92.2 ± 10.7	91.9 ± 12.4	92.1 ± 11.5
hs-CRP (mg/L)	2.4 ± 2.2	3.4 ± 2.7 *	2.9 ± 2.5 *	1.5 ± 1.4	1.7 ± 2.2	1.6 ± 1.8
CX3CL1 (pM/L)	33.8 ± 40.0	35.9 ± 40.0	35.2 ± 39.2 *	18.9 ± 11.2	17.6 ± 8.3	18.3 ± 9.7
CX3CR1 mRNA levels (relative quantification, RQ)	3.3 ± 0.7 *	1.5 ± 1.4 *	2.1 ± 2.5 *	0.2 ± 0.3	0.9 ± 1.9	0.6 ± 1.4
Carotid IMT (mm)	0.764 ± 0.193 *	0.636 ± 0.145 *	0.693 ± 0.178 *	0.535 ± 0.116	0.546 ± 0.094	0.541 ± 0.104
Atheroma plaque (*n* (%))	8 (38.1) *	6 (23.1)	14 (29.8) *	2 (11.1)	3 (15)	5 (13.2)

* *p* < 0.05 between controls and FCH. Apo B: apolipoprotein B; BMI: body mass index; DBP: diastolic blood pressure; FCH: familial combined hyperlipidaemia; HDL-C: high-density lipoprotein cholesterol; HOMA: homeostasis model assessment; hs-CRP: high-sensitivity C-reactive protein; IMT: intima–media thickness; LDL-C: low-density lipoprotein cholesterol; SBP: systolic blood pressure.

**Table 2:** Characteristics of the population according to diagnosis and the presence of insulin resistance.

	IR FCH (*n* = 18)	Non-IR FCH (*n* = 20)	Control (*n* = 38)
Age (years old)	56.4 ± 8.2	59.4 ± 8.5	55.3 ± 8.6
Sex (female/male)	12/18 ^+^	14/3 *	20/18
BMI (kg/m^2^)	32.1 ± 5.4 *	30.3 ± 5.6 *	25.3 ± 3.3
Waist circumference (cm)	103.9 ± 12.8 *	97.4 ± 14.6 *	86.9 ± 9.8
SBP (mmHg)	138.9 ± 11.8 *	135.3 ± 14.9 *	122.2 ± 16.9
DBP (mmHg)	87.4 ± 9.2 *	84.5 ± 8.5 *	74.4 ± 9.8
Glucose (mg/dL)	127.8 ± 34.2 *^+^	98.5 ± 18.3	88.7 ± 8.1
Insulin (μU/mL)	18.8 ± 7.8 *^+^	8.8 ± 2.6	6.1 ± 2.4
HOMA index	5.8 ± 2.6 *^+^	2.1 ± 0.7	1.3 ± 0.5
Total cholesterol (mg/dL)	237.5 ± 42.7 *	227.8 ± 51.0	208.1 ± 23.6
HDL-C (mg/dL)	49.3 ± 9.6 *^+^	58.9 ± 13.6	59.3 ± 11.9
LDL-C (mg/dL)	149.6 ± 28.4	141.4 ± 42.9	134.8 ± 21.1
Triglycerides (mg/dL)	215.0 ± 108.3 *^+^	141.2 ± 50.3 *	82.6 ± 24.4
Apo B (mg/dL)	116.7 ± 21.8 *	105.4 ± 22.3 *	92.1 ± 11.5
hs-CRP (mg/L)	3.2 ± 2.7 *	2.6 ± 2.1	1.6 ± 1.8
CX3CL1 (pM/L)	40.4 ± 43.5 *	25.8 ± 29.9	18.3 ± 9.7
CX3CR1 mRNA levels (relative quantification, RQ)	2.7 ± 2.8 *^+^	0.9 ± 0.6	0.6 ± 1.4
Carotid IMT (mm)	0.738 ± 0.259 *	0.627 ± 0.146	0.541 ± 0.104
Atheroma plaque (*n* (%))	11 (36.7) *^+^	3 (17.6)	5 (13.2)

* *p* < 0.05 between controls ^+^ *p* < 0.05 between non-IR FCH. Apo B: apolipoprotein B; BMI: body mass index; DBP: diastolic blood pressure; FCH: familial combined hyperlipidaemia; HDL-C: high-density lipoprotein cholesterol; HOMA: homeostasis model assessment; hs-CRP: high-sensitivity C-reactive protein; IMT: intima–media thickness; IR: insulin resistant; LDL-C: low-density lipoprotein cholesterol; SBP: systolic blood pressure.

**Table 3:** Characteristics of the population according to diagnosis, the presence of insulin resistance, and sex.

	IR FCH		Non-IR FCH		Control Group	
	Men (*n* = 18)	Women (*n* = 12)	Men (*n* = 3)	Women (*n* = 14)	Men (*n* = 18)	Women (*n* = 20)
Age (years old)	56.5 ± 7.4	58.9 ± 9.3	54.3 ± 7.5	58.3 ± 9.3	53.1 ± 7.6	57.2 ± 9.2
BMI (kg/m^2^)	30.6 ± 3.0 ^≠^¥	35.2 ± 6.7 *	25.3 ± 1.2	30.5 ± 5.9 *	24.8 ± 3.2	25.7 ± 3.4
Waist circumference (cm)	104 ± 9.9 ^≠^	106.9 ± 15.9 *^+^	99.0 ± 8.5	94.2 ± 14.7	88.9 ± 10.0	85.3 ± 9.5
SBP (mmHg)	137.6 ± 9.1 ^≠^	138.9 ± 15.1 *	126.7 ± 8.1	138.9 ± 15.6 *	120.5 ± 15.4	123.7 ± 18.3
DBP (mmHg)	85.2 ± 7.2 ^≠^	89.1 ± 11.4 *	79.1 ± 5.0	87.1 ± 8.8 *	73.6 ± 9.1	75.0 ± 10.5
Glucose (mg/dL)	130.3 ±37.6 ^≠^	124.2 ± 32.8 *	101.7 ± 23.0	99.7 ± 16.5 *	89.3 ± 8.8	88.2 ± 7.7
Insulin (μU/mL)	16.4 ± 6.6 ^≠^¥	21.9 ± 9.1 *	8.3 ± 0.7	9.4 ± 3.3 *	6.2 ± 2.3	6.0 ± 2.6
HOMA index	5.4 ± 2.4 ^≠^¥	6.5 ± 3.0 *	2.1 ± 0.7	2.3 ± 0.9 *	1.4 ± 4.9	1.3 ± 0.6
Total cholesterol (mg/dL)	227.6 ± 41.1 ^≠^	244.8 ± 42.1 *	222.7 ± 64.3	236.0 ± 52.4	203.7 ± 16.7	212.0 ± 28.2
HDL-C (mg/dL)	44.7 ± 6.4 ^≠^	52.9 ± 8.5 *	52.3 ± 24.4	62.5 ± 10.6	55.6 ± 13.4	62.7 ± 9.6
LDL-C (mg/dL)	142.3 ± 21.9	155.7 ± 34.1 *	139.7 ± 46.2	146.4 ± 44.5	134.2 ± 16.4	135.2 ± 24.9
Triglycerides (mg/dL)	223.8 ± 112.5 ^≠^	206.0 ± 106.3 *	153.0 ± 34.8 ^≠^	140.3 ± 58.8 *	80.6 ± 26.2	84.5 ± 23.2
Apo B (mg/dL)	115.3 ± 21.0 ^≠^	117.7 ± 24.6 *	110.0 ± 19.1	106.1 ± 23.5	92.2 ± 10.7	91.9 ± 12.4
hs-CRP (mg/L)	2.5 ± 2.4	4.3 ± 2.9 *	2.1 ± 0.5	2.7 ± 2.3	1.5 ± 1.4	1.7 ± 2.2
CX3CL1 (pM/L)	36.4 ± 35.3 ^≠^	44.3 ± 50.5	28.7 ± 21.9 ^≠^	23.5 ± 10.4	18.9 ± 11.2	17.6 ± 8.3
CX3CR1 mRNA levels (relative quantification, RQ)	3.5 ± 3.3 ^≠^	2.1 ± 1.6 *	0.6 ± 0.4	1.0 ± 0.9	0.2 ± 0.3	0.9 ± 1.9
Carotid IMT (mm)	0.768 ± 0.203 ^≠^	0.675 ± 0.137 *	0.693 ± 0.136	0.602 ± 0.147	0.535 ± 0.116	0.546 ± 0.094
Atheroma plaque (n (%))	7 (38.9) ^≠^	4 (33.3) *	1 (33.3)	2 (14.3)	2 (11.1)	3 (15)

* *p* < 0.05 between FCH and control women; ^+^ *p* < 0.05 between non-IR FCH and IR FCH women; ^≠^ *p* < 0.05 between FCH and control men; ¥ *p* < 0.05 between non-IR FCH and IR FCH men. Apo B: apolipoprotein B; BMI: body mass index; DBP: diastolic blood pressure; FCH: familial combined hyperlipidaemia; HDL-C: high-density lipoprotein cholesterol; HOMA: homeostasis model assessment; hs-CRP: high-sensitivity C-reactive protein; IMT: intima–media thickness; IR: insulin resistant; LDL-C: low-density lipoprotein cholesterol; SBP: systolic blood pressure.

**Table 4:** Correlations between CX3CL1 levels, CX3CR1 expression, and different variables.

	CX_3_CR1 mRNA Levels (Relative Quantification, RQ)		CX_3_CL1 (pM/L)	
	*p* Value	r	*p* Value	r
Age (years old)	0.182	−0.150	0.419	0.106
Sex	0.554	0.067	0.771	−0.038
BMI (kg/m^2^)	0.302	0.116	0.567	0.077
Waist circumference (cm)	0.143	0.17	0.603	0.072
SBP (mmHg)	0.266	0.127	0.493	0.091
DBP (mmHg)	0.079	0.199	0.127	0.201
Glucose (mg/dL)	**0.009**	**0.289**	**<0.001**	**0.407**
Insulin (μU/mL)	**<0.001**	**0.482**	**0.05**	**0.254**
HOMA index	**<0.001**	**0.47**	**0.017**	**0.307**
Total cholesterol (mg/dL)	**0.021**	**0.256**	**0.012**	**0.324**
HDL-C (mg/dL)	**0.009**	**−0.288**	0.664	−0.057
LDL-C (mg/dL)	0.074	0.2	**0.018**	**0.304**
Triglycerides (mg/dL)	**<0.001**	**0.409**	0.126	0.2
Apo B (mg/dL)	**<0.001**	**0.395**	0.128	0.167
hs-CRP (mg/L)	0.267	0.128	0.537	0.083
Carotid IMT (mm)	**<0.001**	**0.403**	0.547	0.079
Atheroma plaque (n (%))	0.854	0.021	**0.048**	**0.257**

Bold indicates statistically significant. Apo B: apolipoprotein B; BMI: body mass index; DBP: diastolic blood pressure; HDL-C: high-density lipoprotein cholesterol; HOMA: homeostasis model assessment; hs-CRP: high-sensitivity C-reactive protein; IMT: intima–media thickness; LDL-C: low-density lipoprotein cholesterol; SBP: systolic blood pressure.

## Data Availability

The data that support the findings of this study are available from the corresponding author upon reasonable request.

## Abbreviations

The following abbreviations are used in this manuscript:

Apo B	Apolipoprotein B
BMI	Body mass index
DBP	Diastolic blood pressure
FCH	Familial combined hyperlipidaemia
HDL-C	High density lipoprotein cholesterol
HOMA	Homeostasis model assessment
hs-CRP	High-sensitivity C-reactive protein
IMT	Intima–media thickness
IR	Insulin resistance
LDL-C	Low-density lipoprotein cholesterol
SBP	Systolic blood pressure
